# Hematological parameters in patients with chronic pain on long-term dipyrone therapy followed in a tertiary hospital: a retrospective cohort study

**DOI:** 10.1016/j.clinsp.2026.101124

**Published:** 2026-09-13

**Authors:** Gibran E. Harcha Munoz, Felipe Chiodini Machado, Heleno de Paiva Oliveira, Maria F. de Paula Ferreira, Áquila Lopes Gouvêa, Hazem Adel Ashmawi

**Affiliations:** a Department of Anesthesia, Hospital das Clínicas, Faculdade de Medicina da Universidade de São Paulo, São Paulo, SP, Brazil; b Universidade Nove de Julho (Uninove), São Paulo, SP, Brazil

**Keywords:** Chronic pain, Dipyrone (Metamizol), Hematologic effects, Agranulocytosis, Pain management

## Abstract

**Background:**

Chronic pain is a significant public health problem, with high prevalence and substantial impact on quality of life and costs in healthcare, particularly in the context of ongoing opioid abuse crises in many countries. Chronic pain management often involves a multimodal approach. Dipyrone or metamizole has been used widely for several decades as a non-opioid analgesic. Despite its effectiveness, concerns regarding potential side effects, such as agranulocytosis, have led to its ban in several countries. This study aimed to describe changes in erythrocyte, leukocyte, neutrophil, and platelet counts among patients with chronic pain receiving dipyrone therapy for longer than six months.

**Methods:**

A retrospective cohort study was conducted using data manually collected from medical records of patients treated at the Pain Outpatient Clinic of a tertiary hospital in Brazil, between 2012 and 2022. The research was registered and approved by the institutional ethical Committee. A total of 1,880 adult patients with chronic pain of different causes who received well documented continuous dipyrone therapy for at least six months. Blood counts before dipyrone use and at the end of dipyrone therapy were evaluated. A control group of chronic pain patients not exposed to dipyrone was not available, which represents a major limitation. Statistical analyses such as paired *t*-tests, Pearson correlations, and multivariate regression were performed to assess changes in blood cell counts, identify any relationships with dipyrone dosage, therapy duration, and other factors.

**Results:**

The mean age of the study population was 58.7 years, with a mean duration of dipyrone therapy of 6.5 years. Statistically significant decreases were observed in neutrophil, leukocyte, and platelet counts from baseline to follow-up; however, these reductions were not clinically relevant and within normal established laboratory reference values. No severe hematological or severe drug reaction such as agranulocytosis were observed. A limited multivariable model did not identify clinically meaningful associations between prescribed dipyrone dose or treatment duration and blood count changes.

**Conclusions:**

In this large cohort of chronic pain patients on long-term dipyrone therapy, only mild and clinically insignificant variations in hematological parameters were observed, and no cases of agranulocytosis were identified. However, the absence of an unexposed control group of chronic pain patients and the lack of systematic data on concomitant medications preclude attributing these observed changes to dipyrone alone.

## Highlights

• A retrospective cohort included 1,880 chronic pain patients prescribed long-term dipyrone.
• Mild blood count variations remained within laboratory reference ranges.
• No cases of agranulocytosis were identified during follow-up.
• Dose and duration showed no clinically meaningful hematologic associations.

## Introduction

Chronic pain is currently considered a public health problem due to its high prevalence. A study conducted in 15 European and Asian countries revealed chronic prevalence ranging from 12% to 30%.^1^ Chronic pain leads to high morbidity and decrease in quality of life, as well as being associated with high costs for healthcare systems. Data showed that in the US, total spending on pain burdens the healthcare system by $560 to $635 billion dollars per year.^2^ Chronic pain has multifactorial causes, related to physical, psychological, environmental, and cultural characteristics. Therefore, its treatment is rarely conducted with monotherapy, necessitating a multimodal approach.^3^

Dipyrone, or metamizole, is a non-narcotic pyrazolone derivative that was first synthesized in 1920, with mass production beginning in 1922. It has analgesic, antipyretic, and spasmolytic effects; however, its mechanisms of action are not fully understood.^4^ This medication is used in the treatment of chronic pain as part of a multimodal approach.

Dipyrone is the most commonly used analgesic in Brazil, both in pediatric and adult populations, with over 100 to 150 million units sold per year.^5^ It is popular due to its relatively low cost and effectiveness. A retrospective cohort study involving over 380,000 patients determined that dipyrone is widely used as a painkiller, either alone or in combination with other drugs.^6^

The literature indicates that dipyrone has good analgesic potency for acute.^7^ and postoperative pain.^8^ It is also frequently used for chronic pain of mild to moderate intensity as part of a multimodal analgesia strategy.^9^

Metamizole is freely available in Spain, Russia, Brazil, Mexico, and Israel, while in Germany it can only be obtained through prescription, where it has become the preferred non-opioid analgesic for perioperative and chronic pain management.^10^

However, several countries, including the USA, Japan, Canada, the UK, and India, have banned the use of dipyrone^11^ due to its alleged side effects, specifically agranulocytosis, which is defined as a neutrophil count of less than 500 µL.^12^

The first reported cases of this side effect emerged in the 1960s,^13^ leading Sweden and other nations to ban dipyrone around 1974 due to concerns about blood dyscrasias. In the following years, more countries joined this prohibition, particularly after the publication of the International Agranulocytosis and Aplastic Anemia Study.^14^

Multiple studies have reported varying risks of agranulocytosis. However, more recent studies have shown an incidence of 0.46 to 1.63 cases per million person-days.^15^ Furthermore, a multicenter and multinational study held in Brazil, Argentina and Mexico during a 4-year period calculated that the incidence of agranulocytosis was 0.38 cases per million person-years (0.35 in Brazil).^16^

On the other hand, the incidence of agranulocytosis related to drugs is estimated in the literature as 3.46:1 million,^17^ although other authors estimated an incidence between 1.6:1 million to 7.0:1 million inhabitants per year.^18^^,^^19^

There are reports on the complications associated with dipyrone, but none with strong statistical value. Therefore, the relevance of this study lies in adding evidence to the literature on dipyrone-induced agranulocytosis in complex patients using dipyrone for long periods.

## Objective

The present study aimed to follow hematological parameters in patients under chronic use of dipyrone, longer than six months, on the variation in the counts of erythrocytes, leukocytes, and platelets in patients with chronic pain in a tertiary university hospital.

### Methods

This study is reported following the STROBE guidelines for observational studies. The complete STROBE checklist is provided as Supplementary Material.^20^ It was a retrospective cohort, the data were obtained from the review of medical records of all the patients treated between 2012 and 2022 at the pain outpatient clinic of the Hospital das Clínicas of the Faculty of Medicine of the University of São Paulo (HCFMUSP) in Brazil, a tertiary hospital that treats highly complex patients.

The research was registered and approved by the Ethical Committee from the Hospital das Clínicas da Faculdade de Medicina da Universidade de São Paulo (approval number 3.491.246), the requirement for individual written informed consent was waived, since data collection was anonymized and indirectly performed from the medical records.

This study retrospectively analyzed data extracted from electronic medical records. For each patient, the following information was collected: Patient Identification Number (PIN), gender, age, dipyrone starts date, dipyrone end date and duration of continuous dipyrone use. Additionally, complete blood counts were analyzed at two time points. The baseline blood count was defined as the last available complete blood count recorded before the first documented dipyrone prescription. The follow-up blood count was defined as the last available complete blood count recorded after at least six months of continuous dipyrone prescription, preferably closest to the end of the documented prescription period. Because this was a retrospective medical record study, the interval between baseline and follow-up blood counts was not predefined and varied substantially among patients. The blood counts included erythrocytes (million/mm^3^), hemoglobin (g/dL), total leukocytes (cells/mm^3^), and platelets (cells/mm^3^).

This study included adult patients (≥ 18-years-old) presenting non cancer chronic pain, without neuropathic pain, that were followed at the pain outpatient clinic of the Central Institute of Hospital das Clínicas da FMUSP between 2012 and 2022 that had used dipyrone for at least 6 months, as documented in their medical records. Patients were excluded if they had incomplete electronic medical records, pre-existing hematologic disorders (including leukopenia, neutropenia, thrombocytopenia, anemia, pancytopenia, or known bone marrow disorders such as myelodysplastic syndromes, aplastic anemia, or leukemia), or a duration of dipyrone use of less than 6 months.

Data were manually extracted from individual medical records by our research team. This process, which spanned over a year due to the high number of patients, involved the meticulous compilation and organization of data within a secure spreadsheet. From this dataset, we determined the duration of documented dipyrone prescription (in months) and the most frequently prescribed daily dose (in grams/day), as recorded in the medical chart.

In cases of significant data discrepancies, a comprehensive review of the corresponding medical record was conducted to identify potential causes. This involved retrieving the complete medical record and independent review by two senior researchers to ensure thoroughness and minimize bias.

To assess changes in blood cell counts, paired t-tests were conducted for erythrocytes, neutrophils, leukocytes, and platelets. Pearson correlation coefficients were calculated to examine the relationships between each cell count and age, duration of dipyrone therapy (days), and dipyrone dosage. Finally, multivariate regression models were developed for each cell type, with dipyrone dosage and duration of therapy included as predictors. All analyses were performed using Google Spreadsheets and R (version 4.4.2). Complete datasets generated and analyzed during this study are available from the corresponding author upon request.

## Results

Table 1 shows demographic characteristics of our study population.

**Table 1:** Demographics, n = 1880.

Male count (%)	589 (31.3%)
**Age, average ± SD**	58.7 ± 15.8
**Days of therapy, average ± SD**	2343 ± 1526
**Ethnicity count (%)**	
White	1388 (73.8%)
Mulato (Mixed-race Brazilian)	170 (9.0%)
Black	148 (7.8%)
Native Brazilian	114 (6.0%)
N.I.	35 (1.8%)

Statistically significant decreases in neutrophil, leukocyte, and platelet counts were observed; however, these reductions are not clinically relevant. Delta values were calculated at the individual patient level as end-of-prescription count minus initial count, and then summarized as mean ± SD. Therefore, the mean delta does not necessarily correspond to the arithmetic difference between the descriptive column means (Table 2).

**Table 2:** Overall cell count and statistical test.

	Delta, average ± SD	Average initial count	Average end of prescription	Paired *t*-test (p-value)
**Erythrocytes (million/mm³)**	0.10 ± 4.4	4.46	5.04	0.31
**Neutrophils (cells/mm³)**	-0.2 ± 3.04	4.78	4.54	<0.001
**Leukocytes (cells/mm³)**	-0.30 ± 3.66	7.67	5.66	<0.001
**Platelets (cells/mm³)**	-12.88 ± 86.4	270	223	<0.001

Initial analysis did not reveal any significant correlation between cell count variation and age, duration of therapy, or dosage when considered independently (Table 3).

**Table 3:** Pearson correlation coefficient between cell counts and possible factors.

	Age	Days of therapy	Dosage
**Erythrocytes**	0.01	0.03	-0.03
**Neutrophils**	0.02	-0.02	0.05
**Leukocytes**	0.03	-0.02	0.05
**Platelets**	0.01	-0.07	0.01

A multivariable linear regression analysis was subsequently performed to assess the potential influence of prescribed dose and treatment duration on observed cell count variation (Table 4).

**Table 4:** Multivariable linear regression of hematologic changes.

Outcome	Predictor	β	95% CI	p-value
Leukocytes	Dosis	0.112	0.005 to 0.220	0.041
	Days of use	-0.000045	-0.000154 to 0.000064	0.418
Neutrophils	Dosis	0.076	-0.012 to 0.165	0.092
	Days of use	-0.000006	-0.000096 to 0.000085	0.904
Platelets	Dosis	1.33	-1.24 to 3.89	0.310
	Days of use	-0.00327	-0.00587 to -0.00068	0.014
Erythrocytes	Dosis	-0.199	-0.381 to -0.017	0.032
	Days of use	0.000151	-0.000033 to 0.000336	0.107

Table 4 All models were adjusted for sex and age. Across all analyses, the models demonstrated very low explanatory power (R²≈0.002–0.004).

Given the multiple comparisons performed, these isolated statistically significant associations should be interpreted with caution and are likely due to chance or unmeasured confounding, particularly considering the negligible effect sizes and R² values.

Across all models presented in Table 4, effect sizes were extremely small, and models explained negligible variance (R²≈0.002–0.004), indicating no clinically meaningful association between dose or duration of use and changes in leukocyte, neutrophil, platelet, or erythrocyte counts.

Although isolated statistically significant associations were observed (e.g., prescribed dose with leukocytes and erythrocytes, and duration with platelets), effect sizes were minimal and inconsistent across outcomes, suggesting that these findings are unlikely to be clinically relevant and may reflect chance, residual variability in a large sample, or unmeasured confounding. In addition, no clear biological gradient was observed.

One death unrelated to dipyrone or a hematologic adverse event was recorded during the follow-up period.

## Discussion

In this retrospective observational study, we examined hematologic parameters in 1,880 adult patients with chronic non-cancer and non-neuropathic pain treated at a tertiary academic hospital in Brazil, all of whom received continuous dipyrone therapy for at least six months. The mean duration of treatment was 6.5 years, representing prolonged exposure. We observed statistically significant declines in leukocyte, neutrophil, and platelet counts over time. However, these changes were very modest in magnitude, and all cell counts remained within established hematimetric normal ranges, indicating no abnormality or illness. The isolated statistically significant regression coefficients observed for some outcomes were small, inconsistent, and explained negligible variance; therefore, they may reflect chance or unmeasured confounding rather than a clinically interpretable dose- or duration-related pattern.

In this large cohort of chronic pain patients on long-term dipyrone therapy, we observed only mild, clinically insignificant variations in hematological parameters, and no cases of agranulocytosis were identified. However, the lack of a control group and data on concomitant medications precludes any attribution of these minor changes to dipyrone alone. Given the tertiary-care setting, the mild statistically significant decreases observed in neutrophil, leukocyte, and platelet counts may reflect the underlying disease burden, intercurrent clinical events, and concomitant medications typical of this complex population rather than a drug-specific effect.

Blood count alterations can occur when certain drugs are administered, which may be considered a hematological adverse drug reaction. Depending on the severity, this can be asymptomatic and detected only through laboratory tests. However, some of these alterations can be quite serious, leading to prolonged hospitalization or even life-threatening conditions. Hematological adverse drug reactions can affect a single cell line, multiple cell lines, or all bone marrow cell lines.^21^ One of the most feared hematological adverse drug reactions is agranulocytosis, inherited or acquired, secondary to autoimmune diseases, bone marrow disorders, cancers that affect the bone marrow, chemotherapy, infections, or prescription drugs. No case of agranulocytosis was observed in the population of the study.

These findings align with prior evidence suggesting that, although dipyrone use may be accompanied by statistically detectable alterations in blood cell profiles, such effects appear clinically insignificant. A systematic review and meta-analysis that included over 70 studies, reported a very low incidence of agranulocytosis linked to dipyrone, particularly in short- to medium-term use. Moreover, a large epidemiological study on drug-induced agranulocytosis estimated an incidence of 4.4 cases per million individuals annually, a rate comparable to that of other commonly prescribed medications [IAAAS, 1986].^21^, ^22^, ^23^, ^24^

Taken together, our data analysis revealed no abnormal laboratorial values in this series of patients, including dosage and duration of therapy with dipyrone in a population with complex medical conditions, which are normally followed in tertiary settings, very different from general healthy population. The mean duration of dipyrone use was approximately 6.5-years, which adds descriptive evidence regarding hematological parameters during long-term exposure.

The prolonged use of dipyrone in this cohort likely reflects its role within multimodal analgesia strategies for chronic pain.^25^ In tertiary care setting, where patients often suffer from persistent pain due to degenerative spine conditions, musculoskeletal disorders, or postoperative syndromes, non-opioid analgesics such as dipyrone are frequently employed for long-term symptom control.^26^ Unlike NSAIDs, dipyrone has a more favorable gastrointestinal and renal profile, and is commonly preferred in Latin America, as supported by both population-based studies and real-world evidence.^27^^,^^28^

A large Brazilian cohort involving over 380,000 patients demonstrated consistent use of dipyrone for chronic pain conditions, without identifying a significant association with severe hematologic events.^26^

Although the underlying mechanisms involving dipyrone-induced agranulocytosis remain incompletely understood, available evidence suggests a primarily idiosyncratic immune-mediated process. Certain HLA haplotypes (e.g., A24-B7, HLA-DQw1) have been implicated in increased susceptibility, but no direct dose-response relationship has been established.^29^

Unlike cytotoxic drugs or some NSAIDs, dipyrone has not been generally characterized as directly myelotoxic, and the absence of clinically meaningful associations between blood count changes and prescribed dose or duration in our study is compatible with the hypothesis of a non-cumulative, immunological etiology in rare cases [30]

As with any observational study, certain limitations need to be considered. The cross-sectional and retrospective nature of our study imposes limitations in assessing causality concerning dipyrone and any hematologic outcomes. A major limitation is the absence of a control group of chronic pain patients not using dipyrone, which prevents attribution of the observed results solely or mainly to dipyrone. Although the sample included nearly 1900 patients, it may still have lacked statistical power to detect rare adverse events such as agranulocytosis. The majority of our sample was formed by chronic pain patients undergoing multimodal analgesic therapy, and other concomitant medications or medical conditions could have independently affected blood cell counts, potentially introducing bias that cannot be fully ruled out. In addition, treatment adherence could not be objectively confirmed, as continuous use was inferred from medical record documentation and patient-reported information. Therefore, the prescribed daily dose may not necessarily reflect the actual dose consumed by each patient.

## Other information

This research did not receive any specific grant from funding agencies in the public, commercial, or non-profit sectors.

## Authors’ contributions

Gibran E. Harcha Munoz: Investigation; writing-original draft; writing-review & editing; visualization.

Felipe Chiodini Machado: Data curation; writing-review & editing; project administration; methodology.

Heleno de Paiva Oliveira: Methodology; investigation; resources; formal analysis.

Maria F de Paula Ferreira: Investigation; data curation.

Áquila Lopes Gouvêa: Project administration; investigation; visualization.

Hazem Adel Ashmawi: Conceptualization; writing-review & editing; supervision.

## Data availability

The datasets generated and/or analyzed during the current study are available from the corresponding author upon reasonable request.

## Declaration of competing interest

The authors declare no conflicts of interest.
