# Supplementary material for: Hematological parameters in patients with chronic pain on long-term dipyrone therapy followed in a tertiary hospital: a retrospective cohort study
Source: Clinics (Sao Paulo). 2026 Sep 13;81:101124. doi: 10.1016/j.clinsp.2026.101124 (PMC13587901; doi:10.1016/j.clinsp.2026.101124)
Supplement: Supplementary file 2 [file mmc2.docx]

**Supplementary Material: STROBE Checklist for Cohort Studies**

Manuscript: Hematological Parameters in Patients with Chronic Pain on Long-Term Dipyrone Therapy followed in a tertiary hospital: A Retrospective Cohort Study

*Checklist used: STROBE Statement — checklist of items that should be included in reports of cohort studies. The table below identifies where each item is addressed in the revised manuscript. N/A = not applicable.*

| **Section / item** | **No.** | **STROBE recommendation** | **Location and reporting details in the revised manuscript** |
| --- | --- | --- | --- |
| **Title and abstract** | **1a** | Indicate the study's design with a commonly used term in the title or the abstract. | Title page/manuscript title: 'Retrospective Cohort Study'. Abstract, Methods: 'A retrospective cohort study was conducted...'. |
| **Title and abstract** | **1b** | Provide in the abstract an informative and balanced summary of what was done and what was found. | Abstract includes Background, Methods, Results, and Conclusions. The conclusions are descriptive and state the lack of a control group and concomitant medication data as limitations to attribution. |
| **Introduction: Background/rationale** | **2** | Explain the scientific background and rationale for the investigation being reported. | Introduction describes chronic pain burden, multimodal analgesia, dipyrone/metamizole use, concerns about agranulocytosis, and the rationale for evaluating hematological parameters during long-term exposure. |
| **Introduction: Objectives** | **3** | State specific objectives, including any prespecified hypotheses. | Objective section states that the study aimed to follow hematological parameters in patients using dipyrone for longer than six months, focusing on erythrocytes, leukocytes, and platelets in patients with chronic pain at a tertiary university hospital. No prespecified hypothesis is stated. |
| **Methods: Study design** | **4** | Present key elements of study design early in the paper. | Methods opening: retrospective cohort study based on medical record review; STROBE reporting statement included. |
| **Methods: Setting** | **5** | Describe the setting, locations, and relevant dates, including periods of recruitment, exposure, follow-up, and data collection. | Methods: Pain Outpatient Clinic, Hospital das Clinicas, Faculty of Medicine, University of Sao Paulo, Brazil; patients treated between 2012 and 2022; exposure defined as documented dipyrone prescription for at least six months; baseline and follow-up blood count definitions provided. The interval between baseline and follow-up blood counts was not predefined and varied among patients. |
| **Methods: Participants** | **6a** | Give the eligibility criteria, and the sources and methods of selection of participants. Describe methods of follow-up. | Methods: adults (>=18 years) with non-cancer chronic pain without neuropathic pain, followed at the pain outpatient clinic between 2012 and 2022, using dipyrone for at least six months as documented in medical records. Exclusions: incomplete electronic medical records, pre-existing blood disorders, or dipyrone use <6 months. Follow-up was retrospective through medical record review and available blood counts. |
| **Methods: Participants** | **6b** | For matched studies, give matching criteria and number of exposed and unexposed. | Not applicable. This was not a matched study and no unexposed control group was available. |
| **Methods: Variables** | **7** | Clearly define all outcomes, exposures, predictors, potential confounders, and effect modifiers. Give diagnostic criteria, if applicable. | Methods/Results define outcomes as hematological parameters from complete blood counts, including erythrocytes, hemoglobin, total leukocytes, neutrophils, and platelets. Exposure was long-term documented dipyrone prescription. Predictors included prescribed daily dipyrone dose and duration of therapy. Regression models were adjusted for sex and age. No effect modifiers were prespecified. |
| **Methods: Data sources/measurement** | **8** | For each variable of interest, give sources of data and details of methods of assessment (measurement). Describe comparability of assessment methods if there is more than one group. | Methods: data were manually extracted from electronic medical records into a secure spreadsheet. Baseline blood count was the last available complete blood count before the first documented dipyrone prescription. Follow-up blood count was the last available complete blood count after at least six months of continuous prescription, preferably closest to the end of the documented prescription period. Prescribed daily dose was recorded from the medical chart. Comparability between groups is not applicable because there was no control group. |
| **Methods: Bias** | **9** | Describe any efforts to address potential sources of bias. | Methods: significant data discrepancies were reviewed by retrieving the complete medical record and through independent review by two senior researchers. Discussion/Limitations explicitly acknowledge retrospective design, lack of control group, unmeasured disease severity, concomitant medications, and inability to objectively confirm adherence. |
| **Methods: Study size** | **10** | Explain how the study size was arrived at. | Methods/Results: the cohort comprised all eligible adult patients from the pain outpatient clinic medical records between 2012 and 2022 who met inclusion criteria and had sufficiently documented dipyrone use for at least six months; final analysed sample was 1,880 patients. No a priori sample size calculation was reported because this was a retrospective cohort based on available records. |
| **Methods: Quantitative variables** | **11** | Explain how quantitative variables were handled in the analyses. If applicable, describe which groupings were chosen and why. | Methods/Results: quantitative variables were treated as continuous variables and summarized mainly as mean +/- SD. Blood count deltas were calculated at the individual-patient level as end-of-prescription count minus initial count and then summarized. No categorization of continuous variables was reported. |
| **Methods: Statistical methods** | **12a** | Describe all statistical methods, including those used to control for confounding. | Methods: paired t-tests for erythrocytes, neutrophils, leukocytes, and platelets; Pearson correlations for blood count variation with age, treatment duration, and prescribed dose; multivariable linear regression models for each cell type with prescribed dose and treatment duration as predictors. Table 4 states that models were adjusted for sex and age and reports beta coefficients, 95% confidence intervals, p-values, and R2 values. |
| **Methods: Statistical methods** | **12b** | Describe any methods used to examine subgroups and interactions. | Not applicable. No subgroup or interaction analyses were reported. |
| **Methods: Statistical methods** | **12c** | Explain how missing data were addressed. | Methods: patients with incomplete electronic medical records were excluded. Results/Table 1 reports an 'N.I.' category for ethnicity. No imputation procedure was reported. |
| **Methods: Statistical methods** | **12d** | If applicable, explain how loss to follow-up was addressed. | Not applicable as a prospective loss-to-follow-up process. Follow-up information was obtained retrospectively from available medical records and available complete blood counts; the non-predefined interval between baseline and follow-up blood counts is acknowledged in Methods. |
| **Methods: Statistical methods** | **12e** | Describe any sensitivity analyses. | Not applicable. No sensitivity analyses were reported. |
| **Results: Participants** | **13a** | Report numbers of individuals at each stage of study - eg numbers potentially eligible, examined for eligibility, confirmed eligible, included in the study, completing follow-up, and analysed. | Abstract/Results: final analysed cohort included 1,880 patients. Methods provide eligibility and exclusion criteria. Detailed numbers screened, excluded, and analysed at each stage were not reported. |
| **Results: Participants** | **13b** | Give reasons for non-participation at each stage. | Methods provide exclusion reasons: incomplete electronic medical records, pre-existing blood disorders, or dipyrone use <6 months. As this was a retrospective medical record study with waived consent, patient non-participation was not applicable; detailed stage-specific exclusion counts were not reported. |
| **Results: Participants** | **13c** | Consider use of a flow diagram. | Not included. Final cohort size and eligibility criteria are reported in the manuscript. |
| **Results: Descriptive data** | **14a** | Give characteristics of study participants (eg demographic, clinical, social) and information on exposures and potential confounders. | Results/Table 1 reports sex, age, days of therapy, and ethnicity. Methods/Results describe dipyrone exposure duration and prescribed daily dose variables. Age and sex were used for adjustment in Table 4. |
| **Results: Descriptive data** | **14b** | Indicate number of participants with missing data for each variable of interest. | Results/Table 1 includes non-informed ethnicity (N.I. = 35, 1.8%). Methods state that incomplete electronic medical records were excluded. A detailed per-variable missing-data table was not reported. |
| **Results: Descriptive data** | **14c** | Summarise follow-up time (eg, average and total amount). | Abstract/Results/Table 1/Discussion report mean duration of dipyrone therapy as 2,343 +/- 1,526 days, approximately 6.5 years. |
| **Results: Outcome data** | **15** | Report numbers of outcome events or summary measures over time. | Results/Table 2 reports initial and end-of-prescription blood counts, individual-level mean deltas +/- SD, and paired t-test p-values for erythrocytes, neutrophils, leukocytes, and platelets. Results state that no severe hematologic event or agranulocytosis was identified and that one death unrelated to dipyrone or a hematologic adverse event was recorded. |
| **Results: Main results** | **16a** | Give unadjusted estimates and, if applicable, confounder-adjusted estimates and their precision (eg, 95% confidence interval). Make clear which confounders were adjusted for and why they were included. | Results/Table 2 reports paired comparisons. Results/Table 4 reports multivariable regression beta coefficients, 95% confidence intervals, p-values, and notes adjustment for sex and age. The text states that isolated statistically significant associations were small, inconsistent, explained negligible variance, and showed no clear biological gradient. |
| **Results: Main results** | **16b** | Report category boundaries when continuous variables were categorized. | Not applicable. Continuous variables were not categorized for the reported analyses. |
| **Results: Main results** | **16c** | If relevant, consider translating estimates of relative risk into absolute risk for a meaningful time period. | Not applicable. The study did not estimate relative risks; it reported longitudinal blood count changes and linear regression estimates. |
| **Results: Other analyses** | **17** | Report other analyses done - eg analyses of subgroups and interactions, and sensitivity analyses. | Results/Table 3 reports Pearson correlations. Results/Table 4 reports multivariable regression models. No subgroup, interaction, or sensitivity analyses were reported. |
| **Discussion: Key results** | **18** | Summarise key results with reference to study objectives. | Discussion opening paragraphs summarize statistically significant but clinically insignificant changes, absence of agranulocytosis, and lack of a clinically interpretable dose- or duration-related pattern. |
| **Discussion: Limitations** | **19** | Discuss limitations of the study, taking into account sources of potential bias or imprecision. Discuss both direction and magnitude of any potential bias. | Discussion/Limitations: retrospective design, lack of unexposed control group, inability to attribute findings to dipyrone alone, lack of concomitant medication data, complex tertiary-care disease burden, limited power for rare agranulocytosis events, and inability to objectively confirm adherence are discussed. Potential direction and magnitude of bias are discussed qualitatively rather than quantitatively. |
| **Discussion: Interpretation** | **20** | Give a cautious overall interpretation of results considering objectives, limitations, multiplicity of analyses, results from similar studies, and other relevant evidence. | Discussion/Conclusion framing: findings are interpreted descriptively as mild, clinically insignificant variations in hematological parameters during long-term dipyrone therapy, with no attribution to dipyrone alone because of the lack of a control group and concomitant medication data. Isolated statistically significant regression findings are interpreted cautiously in light of small effects, negligible variance, chance, residual variability, unmeasured confounding, and lack of biological gradient. Prior literature on agranulocytosis and metamizole/dipyrone is discussed. |
| **Discussion: Generalisability** | **21** | Discuss the generalisability (external validity) of the study results. | Discussion emphasizes that the population consisted of complex chronic pain patients in a tertiary-care setting and was very different from a generally healthy population; results should be interpreted in that clinical context. |
| **Other information: Funding** | **22** | Give the source of funding and the role of the funders for the present study and, if applicable, for the original study on which the present article is based. | Other information: 'This research did not receive any specific grant from funding agencies in the public, commercial, or non-profit sectors.' |

Reference: von Elm E, Altman DG, Egger M, Pocock SJ, Gotzsche PC, Vandenbroucke JP; STROBE Initiative. The Strengthening the Reporting of Observational Studies in Epidemiology (STROBE) statement: guidelines for reporting observational studies. PLoS Med. 2007;4(10):e296. doi:10.1371/journal.pmed.0040296
